# A *Medicago truncatula* NADPH oxidase is involved in symbiotic nodule functioning

**DOI:** 10.1111/j.1469-8137.2010.03509.x

**Published:** 2011-01

**Authors:** Daniel Marino, Emilie Andrio, Etienne G J Danchin, Elodie Oger, Sébastien Gucciardo, Annie Lambert, Alain Puppo, Nicolas Pauly

**Affiliations:** Interactions Biotiques et Santé Végétale, UMR Université de Nice-Sophia Antipolis – INRA 1301 – CNRS 6243, 400 Route des Chappes,dBP 167, F-06903 Sophia Antipolis Cedex, France

**Keywords:** hypoxia, *Medicago truncatula–Sinorhizobium meliloti* symbiosis, NADPH oxidase, nitrogen fixation, nitrogenase, nodule, reactive oxygen species, respiratory burst oxidase homologue

## Abstract

- The plant plasma membrane-localized NADPH oxidases, known as respiratory burst oxidase homologues (RBOHs), appear to play crucial roles in plant growth and development. They are involved in important processes, such as root hair growth, plant defence reactions and abscisic acid signalling.
- Using sequence similarity searches, we identified seven putative RBOH-encoding genes in the *Medicago truncatula* genome. A phylogenetic reconstruction showed that *Rboh* gene duplications occurred in legume species. We analysed the expression of these *MtRboh* genes in different *M. truncatula* tissues: one of them, *MtRbohA*, was significantly up-regulated in *Sinorhizobium meliloti*-induced symbiotic nodules.
- *MtRbohA* expression appeared to be restricted to the nitrogen-fixing zone of the functional nodule. Moreover, using *S. meliloti bacA* and *nifH* mutants unable to form efficient nodules, a strong link between nodule nitrogen fixation and *MtRbohA* up-regulation was shown. *MtRbohA* expression was largely enhanced under hypoxic conditions. Specific RNA interference for *MtRbohA* provoked a decrease in the nodule nitrogen fixation activity and the modulation of genes encoding the microsymbiont nitrogenase.
- These results suggest that hypoxia, prevailing in the nodule-fixing zone, may drive the stimulation of *MtRbohA* expression, which would, in turn, lead to the regulation of nodule functioning.

## Introduction

It is now well established that plants generate reactive oxygen species (ROS) as signalling molecules to control various cellular mechanisms (Neill *et al.*, 2002). Indeed, accumulating experimental evidence shows that ROS are key players in fundamental processes, such as cellular growth (Foreman *et al.*, 2003), stomatal closure (Pei *et al.*, 2000) and plant defence against pathogens (Apel & Hirt, 2004). Moreover, ROS are known to orchestrate plant gene expression (Neill *et al.*, 2002; Vanderauwera *et al.*, 2005), as well as to modulate the activity of key signalling components, such as mitogen-activated protein (MAP) kinases (Rentel *et al.*, 2004).

The involvement of ROS in the legume–rhizobia symbiotic interaction has also been highlighted (Pauly *et al.*, 2006). Legumes are the only plant family with the ability to establish a symbiotic interaction with soil bacteria, commonly named rhizobia, leading to the formation of a new organ, the root nodule, whose primary function is dinitrogen (N_2_) fixation. Nodule formation implicates extensive recognition by both partners in order to allow both an organized journey of the bacteria through the plant, and cell division and differentiation processes leading to the development of the nodule meristem. Finally, nodules will be colonized by bacteria released from infection threads formed on infection (Long, 2001; Oldroyd & Downie, 2008).

The production of ROS has been evidenced in both functional nodules and during the early steps of the interaction. Hydrogen peroxide (H_2_O_2_) has been detected in mature 6-wk-old nodules, mainly in the cell walls of infected cells and also in some infection threads around bacteria (Santos *et al.*, 2001; Rubio *et al.*, 2004). In the early stages, superoxide anion (O_2_^−^) is detected in infection threads (Santos *et al.*, 2001). More recently, the generation of ROS in the cortical cells of *Medicago truncatula* roots after inoculation with *Sinorhizobium meliloti* has been observed *in vivo*, using the 2′,7′-dichlorofluorescein probe (Peleg-Grossman *et al.*, 2007). The importance of ROS production was confirmed using a catalase-overexpressing *S. meliloti* strain, acting as an H_2_O_2_ sink, which showed delayed nodulation (Jamet *et al.*, 2007). Thus, it appears that ROS are essential for optimal symbiosis establishment, and that they are produced as a specific response to infection associated with the nodule developmental programme, rather than as an oxidative burst similar to that encountered in pathogenic systems (Pauly *et al.*, 2006). Moreover, the use of diphenylene iodonium (DPI), which inhibits flavoproteins, such as the gp91^phox^ catalytic subunit of NADPH oxidases (NOXs), abolished ROS production and also suppressed root hair curling and infection thread formation (Lohar *et al.*, 2007; Peleg-Grossman *et al.*, 2007). This strongly suggests the involvement of *M. truncatula* NOX(s) in such ROS production (Peleg-Grossman *et al.*, 2007).

The plant plasma membrane-localized NOXs are homologous to the catalytic subunit (gp91^phox^) of mammalian phagocyte NOXs (Sagi & Fluhr, 2001). Plant NOXs are known as respiratory burst oxidase homologues (RBOHs). RBOHs are transmembrane proteins composed of six transmembrane domains supporting two haem groups, FAD and NADPH hydrophilic domains in the C-terminal region and two calcium-binding domains (EF-hand) in the N-terminal region. NADPH acts as a cytosolic electron donor to the extracellular O_2_ electron acceptor, which is reduced to O_2_^–^ via FAD and two independent haems (Sagi & Fluhr, 2001). Arabidopsis contains 10 RBOH homologues (Sagi & Fluhr, 2006). Microarray data compiled in Genevestigator showed their distribution into three classes: *AtRbohD* and *AtRbohF*, which are expressed in all plant parts, *AtRbohA-G* and *AtRboh I*, which are expressed in the roots, and *AtRbohH* and *AtRbohJ*, specifically expressed in pollen (https://www.genevestigator.com).

Plant RBOHs play crucial roles in plant health and metabolism. *AtRbohD* and *AtRbohF* are involved in ROS-dependent abscisic acid (ABA) signalling and guard cell ABA signal transduction (Kwak *et al.*, 2003). The *Arabidopsis thaliana rhd2* mutant lacking a functional AtRBOHC is root hair defective, thus underlining the role of these proteins in ROS-mediated plant cell growth (Foreman *et al.*, 2003). RBOHs also appear to play important roles in pathogenic plant–microbe interactions. *AtRbohD* and *AtRbohF* are required for full ROS production observed during incompatible interactions with the bacterial pathogen *Pseudomonas syringae* pv *tomato* DC3000 (*avrRpm1*) and the phytopathogenic oomycete *Hyaloperonospora parasitica* (Torres *et al.*, 2002); *NtRbohD* is involved in ROS production in cryptogein-elicited tobacco cells (Simon-Plas *et al.*, 2002). Moreover, during the tobacco response to *Phytophthora infestans* oomycete, *NtRbohA* and *NtRbohB* have been shown to be required for ROS accumulation (Yoshioka *et al.*, 2003). Moreover, a role of NOXs in oxygen-sensing processes has been suggested (Jones *et al.*, 2000; Bailey-Serres & Chang, 2005).

In this framework, there is a need to characterize the roles of RBOHs in the legume–rhizobia symbiotic interaction and, to our knowledge, this is the first analysis on *Rboh* genes in legume nodules. In this work, we describe the phylogenetic analysis and expression profiles of *M. truncatula Rboh* genes, and point out the importance of one RBOH for nodule functioning.

## Materials and Methods

### Plant growth and bacterial strains

*Medicago truncatula* Gaertn. cv Jemalong J6 was used throughout the experiments. Surface-sterilized seeds were placed on 0.4% agar plates in the dark for 1 d at 4°C and then for 3 d at 14°C. Germinated seeds were transferred into either nitrogen-free modified Fahraeus agar plates (root hair isolation) or 1 : 2 sand : vermiculite pots. One week after transfer, axenic plants were inoculated with 200 μl of *S. meliloti* 2011 suspension (OD_600_ = 0.05) per root and nonaxenic plants with 10 ml per pot. Plants in pots were irrigated twice a week with a nitrogen-free nutrient solution (Rigaud & Puppo, 1975). The chamber conditions were 25°C : 22°C day : night, 75% hygrometry, 200 μmol m^−2^ s^−1^ light intensity and a 16 h : 8 h light : dark photoperiod.

For *MtRbohA* expression analysis in nonfixing nodules, *S. meliloti* 1021 *nifH* (Ruvkun *et al.*, 1982) and 1021 *bacA* (Glazebrook *et al.*, 1993) mutants were used.

For hypoxia treatment, 4-wk-old inoculated or control plants were waterlogged with O_2_-deprived nutrient solution for 24 h. Nodules and root tissues were harvested and *MtRboh* gene expression analysis was performed. The efficiency of hypoxia treatment was evaluated by analysing the up-regulation of pyruvate decarboxylase *Medtr2g019000* gene expression (data not shown).

### Identification and phylogenetic analysis of *Rboh* sequences

*Rboh* sequences were retrieved via a similarity search using BlastP (Altschul *et al.*, 1997) with Arabidopsis Rboh sequences as queries against different plant protein sequence databases: http://www.medicago.org/ for *M. truncatula*, http://www.kazusa.or.jp/lotus/ for *Lotus japonicus*, http://rice.plantbiology.msu.edu/ for rice and http://www.phytozome.com/ for all the other already sequenced plant genomes (Supporting Information, Table S1). Multiple sequence alignment was performed using the Muscle program (Edgar, 2004) with standard parameters. The alignment was visually examined and edited for the elimination of sequences that were too short and the removal of alignment columns with too many gaps. Phylogenetic analyses were performed using a maximum likelihood (ML) approach with PhyML (Guindon & Gascuel, 2003) and a Bayesian approach with MrBayes (Ronquist & Huelsenbeck, 2003). ML phylogeny was performed with the LG model of evolution; a gamma distribution of variable substitution rates and a proportion of invariable sites were evaluated from the data by the software, and an approximate likelihood ratio test (aLRT) was launched to evaluate the robustness of the nodes. Bayesian phylogenetic reconstruction was performed with a mixed model of evolution and an evaluation of the gamma distribution and proportion of invariable sites. Congruence was reached with a total of 100 000 generations. Phylogenetic trees were visualized and annotated using FigTree (http://www.tree.bio.ed.ac.uk/software/figtree/).

### Construction of a binary vector for hairy root transformation

For *MtRboh* promoter transcriptional fusions, fragments of *c.* 2000 bp upstream of the start codon were amplified by PCR using the primers indicated in Table S2. Each PCR fragment was first cloned into the pDONR207 donor vector and then into the plant expression vector pKGWFS7 (Karimi *et al.*, 2002) using Gateway technology (Invitrogen, http://www.invitrogen.com).

For the RNA interference (RNAi) construct, the constitutive cauliflower mosaic virus (CaMV) 35S promoter in the pK7GWIWG2D(II) vector (Karimi *et al.*, 2002) was replaced by the nodule-specific *MtNCR001* promoter (E. Boncompagni, unpublished). The fragment for *MtRbohA* inactivation was amplified with 5′-GTGGTACCACTGTGGAGCACATGGATTG-3′ and 5′-CAGCTCGAGCAAAAAGCCTTGGTCTTACAG-3′ as forward and reverse primers, respectively, and cloned by restriction (*Kpn*I and *Xho*I) into pENTR4 (Invitrogen, http://www.invitrogen.com). This fragment, corresponding to the 3′-untranslated region (3′-UTR) of the *MtRbohA* gene, was then cloned into pK7GWIWG2D(II) containing the *MtNCR001* promoter using Gateway technology (Invitrogen, http://www.invitrogen.com). The constructs were checked by DNA sequencing, introduced by electroporation into *Agrobacterium rhizogenes* strain ARqua1 and used for *M. truncatula* root transformation as described previously (Boisson-Dernier *et al.*, 2001).

### Histochemical localization of β-glucuronidase (GUS) activity

GUS activity was assayed histochemically from the nodulated roots of composite plants fixed at −20°C in 90% acetone for 60 min and incubated overnight in 0.5 mM K_3_Fe(CN)_6_, 1 mM K_4_Fe(CN)_6_, 0.8 mM 5-bromo-4-chloro-3-indolyl-β-d-glucuronic acid (X-Gluc, Eurogentec, http://www.eurogentec.com), 0.1 M potassium phosphate buffer, pH 7. Eighty-micrometre-thick vibroslices, obtained with a HM560V Vibratome (Microm, http://www.microm.de) after embedding plant material in 4.5% low-melting-point agarose, were visualized with a Zeiss Axioplan 2 microscope (Carl Zeiss, http://www.zeiss.com) using dark-field optics.

### Total RNA isolation, reverse transcription (RT) and gene expression analysis

Two hundred milligrams of plant material (roots, root hairs, nodules, flowers, pods, shoots, leaves) were ground in liquid nitrogen and total RNA was isolated using Trizol Reagent (Invitrogen, http://www.invitrogen.com). Root hairs were obtained as described previously (Sauviac *et al.*, 2005). The integrity of total RNA was checked on agarose gel and its quantity, as well as purity, was determined spectrophotometrically. Two micrograms of RNA were used as a template for RT reaction in a reaction volume of 20 μl using the Omniscript RT Kit (Qiagen, http://www.qiagen.com) with oligodT or random primers (Invitrogen, http://www.invitrogen.com) for plant and bacterial genes, respectively. Quantitative real-time RT-PCR was carried out using the qPCR Mastermix Plus for SYBR Green I reagent (Eurogentec, http://www.eurogentec.com). Reactions were run on the Chromo4 Real-Time PCR Detection System (Bio-Rad, http://www.bio-rad.com), and quantification was performed with Opticon Monitor analysis software v. 3.1 (Bio-Rad, http://www.bio-rad.com). Every reaction was set up in three technical replicates. The PCR programme used was as follows: polymerase activation (95°C for 5 min), amplification and quantification cycles repeated 40 times (94°C for 15 s, 60°C for 1 min) and melting curve (40 to 95°C with one fluorescence read every 0.5°C). The plant mRNA levels were normalized against two endogenous controls: *40S Ribosomal Protein S8* (TC137982) and *Mtc27* (TC132510) (Van de Velde *et al.*, 2006). The *Smc00324* housekeeping gene was used to normalize the bacterial mRNA levels (Becker *et al.*, 2004). *NifH*/*D* primers were designed as described previously (Naya *et al.*, 2007). The following formula was used for the relative expression ratio calculation: 2^−ΔCT^, with ΔCT = CTgene of interest–CThousekeeping gene. For each experiment, the stability of the reference genes across samples was tested using geNorm software (Vandesompele *et al.*, 2002). The absence of contamination with genomic DNA was tested by quantitative RT-PCR in all RNA samples, before RT. The gene-specific primers used are listed in Table S2.

### Determination of acetylene reduction activity

Nitrogen fixation was determined using the acetylene reduction assay as described previously (Hardy *et al.*, 1968). Nodulated roots from each composite plant (control or RNAi) were placed in 30 ml glass flasks filled with an acetylene–air mixture (C_2_H_2_ : air = 1 : 10 v/v). After 1 h of incubation at 25°C, the amount of ethylene in the gas phase was determined by gas chromatography using a 6890N Network GC system (Agilent Technologies, http://www.agilent.com).

## Results

### Identification, annotation and phylogenetic analysis of *M. truncatula Rboh* genes

Ten *Rboh* genes are present in the *A. thaliana* genome (Sagi & Fluhr, 2006). Using protein sequence similarity search tools with Arabidopsis sequences as queries, we found seven RBOH-encoding genes in the *M. truncatula* genome (http://www.medicago.org). According to their localization in the *M. truncatula* genome and the widely used nomenclature (Torres & Dangl, 2005), we named these genes *MtRbohA–G*. For five of them (*MtRbohA*, *B*, *E–G)*, expressed sequence tags (ESTs) are available in the ‘TIGR *M. truncatula* Gene Index’, and, for four isoforms (*MtRbohA*, *B*, *E* and *G*), tentative consensus sequences have been proposed from EST contigs (Table 1).

**Table 1:** *Medicago truncatula* respiratory burst oxidase homologue (*Rboh*) genes

Gene	Name IMGAG^a^	Genbank^b^ accession number	ORF length (bp)	Number of ESTs	TC number (TIGR)^c^	Affymetrix Probeset^d^
*MtRbohA*	Medtr1g099800	–	2658	7	TC112710	Mtr.39812.1.S1_s_at Mtr.32104.1.S1_s_at
*MtRbohB*	Medtr3g151540	–	2772	6	TC123192, TC123112	Mtr.2439.1.S1_at Mtr.45354.1.S1_at
*MtRbohC*	Medtr3g151570	–	2754	–	–	–
*MtRbohD*	Medtr3g151600	–	2109	–	–	–
*MtRbohE*	Medtr4g144710	AY821801	2799	33	TC126164	Mtr.17607.1.S1_at
*MtRbohF*	Medtr7g067680	–	2550	3	–	Mtr.27053.1.S1_at
*MtRbohG*	Medtr7g138940	AY821802	2688	47	TC112621	Mtr.43415.1.S1_s_at Mtr.32307.1.S1_at Mtr.32307.1.S1_s_at

The International Medicago Genome Annotation Group (IMGAG) (http://www.medicago.org).

Genbank (http://www.ncbi.nlm.nih.gov).

Designation of tentative consensus (TC) from TIGR (http://www.tigr.org). ORF, open reading frame; EST, expressed sequence tag.

Affymetrix Probeset (http://bioinfo.noble.org/gene-atlas/v2/).

Six of the *Rboh* genes (*MtRbohA–C*, *E–G*) have open reading frames (ORFs) of between 2550 and 2790 bp. Analysis of the domain composition of the corresponding encoded proteins (PFAM; Bateman *et al.*, 2004) showed the presence of five typical domains of plant NOXs [respiratory burst NOX domain (PF08414); EF hand (PF00036); ferric reductase-like transmembrane component (PF01794); FAD-binding domain (PF08022); and a ferric reductase NAD-binding domain (PF08030)]. By contrast, the *MtRbohD* sequence has a shorter ORF of 2109 bp, corresponding to a truncated protein lacking the C-terminal ferric reductase NAD-binding domain. However, *c.* 5 kb downstream of the *MtRbohD* stop codon, there is a predicted sequence (TC130541) which corresponds to the lacking ferric reductase NAD-binding domain. Within this 5 kb region, we found a sequence encoding a ‘putative nonlong terminal repeat retroelement reverse transcriptase’, suggesting a retrotransposon insertion within the theoretical *MtRbohD* ancestral sequence. The available data do not provide any evidence to allow the confirmation that *MtRbohD* is able to encode the five-domain full-length protein.

**Figure 1:**
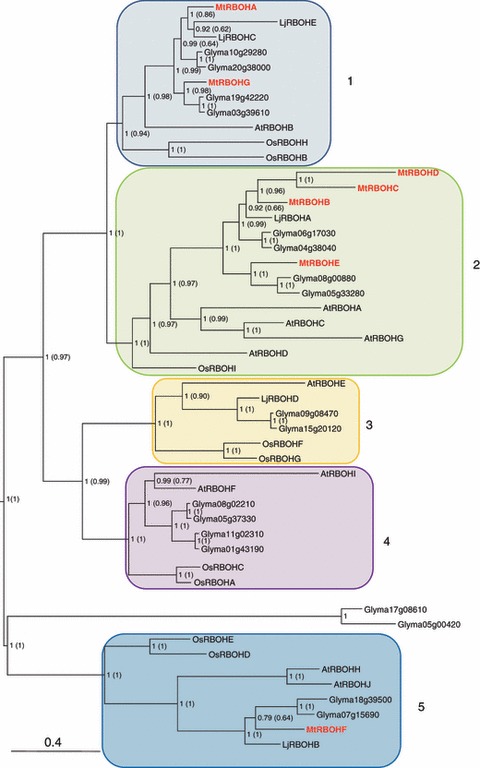
Phylogenetic tree of respiratory burst oxidase homologue (RBOH) amino acid sequences. Five plant species were used for the analysis. Species’ name abbreviations are used as prefixes as follows: Glyma, *Glycine max*; Os, *Oryza sativa*; At, *Arabidopsis thaliana*; Lj, *Lotus japonicas*; Mt, *Medicago truncatula*. The Bayesian tree topology is used as reference in this figure with midpoint rooting. Posterior probability values are indicated at each node and maximum likelihood approximate likelihood ratio test (aLRT) values are shown in parentheses. *Medicago truncatula Rboh* genes are indicated in Table 1. AtRBOHA (At5g07390); AtRBOHB (At1g09090); AtRBOHC (At5g51060); AtRBOHD (At5g47910); AtRBOHE (At1g19230); AtRBOHF (At1g64060); AtRBOHG (At4g25090); AtRBOHH (At5g60010); AtRBOHI (At4g11230); AtRBOHJ (At3g45810); Glyma01g43190; Glyma03g39610; Glyma04g38040, Glyma05g37330; Glyma05g33280; Glyma05g00420; Glyma06g17030; Glyma07g15690; Glyma08g00880; Glyma08g02210; Glyma09g08470; Glyma10g29280; Glyma11g02310; Glyma15g20120; Glyma17g08610; Glyma18g39500; Glyma19g42220; Glyma20g38000; LjRBOHA (CM0094.200); LjRBOHB (CM0147.340); LjRBOHC (CM0299.380); LjRBOHD (CM0013.340); LjRBOHE (CM0147.360); OsRBOHA (Os01g53294); OsRBOHB (Os01g25820); OsRBOHC (Os05g45210); OsRBOHD (Os05g38980); OsRBOHE (Os01g61880); OsRBOHF (Os08g35210); OsRBOHG (Os09g26660); OsRBOHH (Os12g35610); OsRBOHI (Os11g33120).

The seven MtRBOH protein sequences exhibit 47–69% similarity (Table S3) and were used to build phylogenetic trees (Figs 1, S1). There are nine complete *Rboh* genes in the rice (*Oryza sativa*) genome (Wong *et al.*, 2007), five in the not yet fully sequenced genome of *L. japonicus* and 18 in soybean (*Glycine max*). Phylogenetic reconstructions of these plant RBOH proteins, together with those of *A. thaliana* and *M. truncatula*, converged in producing a highly supported topology with both Bayesian and ML approaches, as illustrated by posterior probability and aLRT values. A total of five groups of orthologues can be defined from the phylogenetic tree (Fig. 1). Groups 1, 2 and 5 contain representatives from all the selected species. Interestingly, no *M. truncatula* orthologues are found in groups 3 and 4, which are closely related on the phylogenetic tree. This may reflect incompleteness or gaps in the current version of the *M. truncatula* genome. Group 3 contains representatives of all the other species and group 4 contains all others apart from *L. japonicus* (Fig. 1).

### *MtRboh* expression exhibits specific localization profiles

**Figure 2:**
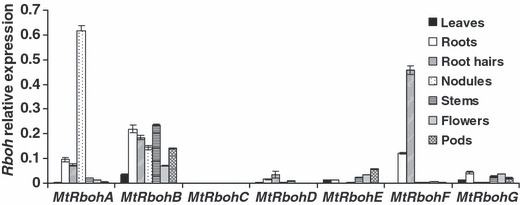
*MtRboh* gene expression analysis in different *Medicago trunculata* tissues. RNA from root hairs (10-d-old seedlings), leaves, roots, nodules, stems (5-wk-old plants), flowers and pods (7-wk-old plants) were used for quantitative real-time RT-PCR analysis. Values were normalized against *40S Ribosomal Protein S8* and *Mtc27* gene expression, which were used as housekeeping genes. Samples were obtained by pooling tissues of 10 plants, and the values are representative of three independent biological replicates. Error bars, +SE.

To characterize the expression profile of *M. truncatula Rboh* genes, we analysed their transcript abundance by quantitative real-time PCR in different plant tissues (leaves, roots, root hairs, nodules, stems, flowers and pods). *MtRbohB* is notably expressed in all the analysed tissues, *MtRbohE* and *G* exhibit low expression levels in all tested tissues and *MtRbohF* is significantly up-regulated in roots and root hairs (Fig. 2). *MtRbohC* and *MtRbohD*, which are highly similar at the amino acid level (*c.* 69%, Table S3) and are grouped in the same phylogenetic cluster (Fig. 1), presented a very low expression level in all tissues examined, which is in agreement with the lack of ESTs in the databases for both genes. The most striking result lies in the remarkable up-regulation of *MtRbohA* expression in nodules. Indeed, *MtRbohA* showed a six-fold higher expression level in nodules than in roots, and four-fold higher than *MtRbohB*, the second most expressed *MtRboh* in nodules (Fig. 2). Our expression profiles are mostly consistent with the already available transcriptome analysis (Benedito *et al.*, 2008) (Fig. S2). The only difference concerned *MtRbohE*, which is detected at the same level in roots and leaves in our conditions, although it was weakly expressed in leaves (Fig. S2).

**Figure 3:**
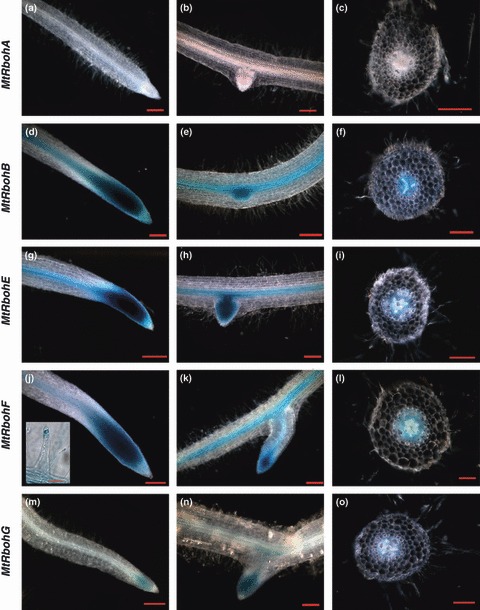
Histochemical analysis of *MtRboh* expression in *Medicago truncatula* roots. Main root tip (a, d, g, j, m), central cortex and secondary apex (b, e, h, k, n), root hair (inset in j) and root transverse section (c, f, i, l, o) from 3-wk-old composite plants. *MtRbohA* (a–c), *MtRbohB* (d–f), *MtRbohE* (g–i), *MtRbohF* (j–l), *MtRbohG* (m–o). Bars, 200 μm (50 μm in inset of j). *n* > 20.

To localize more specifically root tissue expression, histochemical staining was performed using a promoter GUS transcriptional fusion approach. An approx. 2 kb promoter region was chosen (Table S2). As a result of their very low expression levels, *MtRbohC* and *D* were not included in this study. Main root tip (division and elongation zones) and central cylinder staining was observed in the root systems for *MtRbohB*, *E–G* (Fig. 3d,g,j,m). Secondary root meristems were also stained for these genes (Fig. 3e,h,k,n). *MtRbohB, E* and *F* root GUS staining was very strong compared with that of *MtRbohG*. All of these *MtRboh* genes (*B*, *E*, *F*, *G*) showed the same localization. These staining conditions did not reveal *MtRbohA* expression in roots (Fig. 3a–c), although it was detected in the quantitative real-time PCR experiments (Fig. 2). However, when the staining time was increased to 16 h, slight *MtRbohA* expression was observed in vascular tissues (data not shown). An examination of semi-thin transverse sections revealed that the promoter fusion of *MtRbohB*, *E* and *F* directed strong GUS expression, which was restricted to the phloem and the surrounding parenchyma (Fig. 3f,i,l,o). Considering that several authors have found endogenous GUS-like activity in different plant tissues, including *M. truncatula* cv Jemalong (Journet *et al.*, 2001), we assayed either transformed control plant (empty vector) or nontransformed plants, and no background staining was detected for the considered incubation time (data not shown). *MtRbohF* shows a very high expression level in roots, more than 200-fold higher than in any other tissue. Moreover, its expression in root hairs was four-fold higher than in the rest of the roots (Fig. 2). We also detected *MtRbohF* expression in root hairs (Fig. 3j, inset).

**Figure 4:**
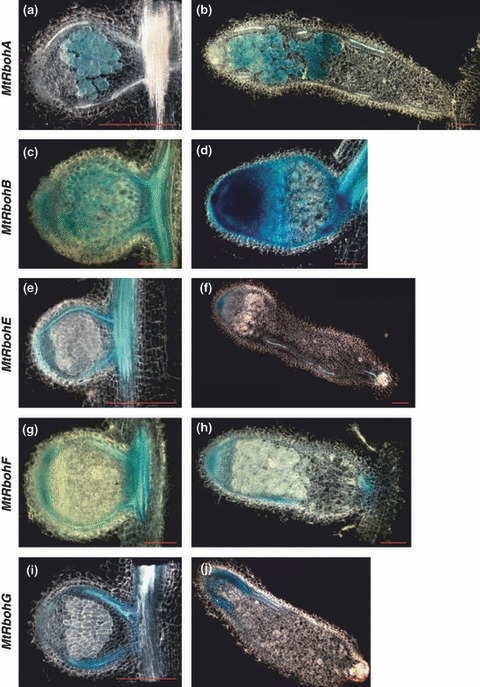
Histochemical analysis of *MtRboh* expression in nodules of *Medicago truncatula* inoculated with *Sinorhizobium meliloti*. Nodules at 12 d post-inoculation (a, c, e, g, i) and 5–7 wk post-inoculation (b, d, f, h, j). *MtRbohA* (a, b), *MtRbohB* (c, d), *MtRbohE* (e, f), *MtRbohF* (g, h), *MtRbohG* (i, j). Bars, 200 μm. *n* > 20.

We analysed *Rboh* gene expression during *M. truncatula* interaction with its microsymbiont. To investigate the cellular localization of *MtRboh* promoter activity during this process, composite transgenic plants were inoculated with *S. meliloti* and longitudinal sections of the nodules were assayed. In 12-d-old root nodules, *MtRbohE–G* GUS staining was detected in vascular bundles, which are a continuation of the root central cylinder already shown to be coloured (Fig. 4e,g,i). GUS coloration was also apparent in the apical region corresponding to the permanent meristem, which is characteristic for indeterminate nodules; no expression was observed in any other zone of the nodule. *MtRbohB* promoter activity, in good agreement with the quantitative real-time PCR results, showed a ubiquitous expression in the nodule (Fig. 4c).

Interestingly, *MtRbohA* expression appears to be restricted to the central tissue of the root nodule (Fig. 4a,b). The limitation of its expression to the infection zone was confirmed by the use of an *S. meliloti* 2011 strain expressing a constitutive *hemA::lacZ* construct (Leong *et al.*, 1985), which allowed the colocalization of *MtRbohA* GUS expression with lacZ staining (Fig. S3). In older nodules (5–7 wk post-inoculation), in which indeterminate nodule zonation is evident, none of the analysed *MtRboh* promoters generated GUS staining in the senescence zone (Fig. 4b,d,f,h,j).

In addition, the GUS staining of 12-d-old nodules confirmed the restricted *MtRbohA* expression to the nodule nitrogen-fixing zone where cells are infected and the nitrogen fixation process takes place (Fig. 4a). Taken together, the results obtained point to a possible role of *MtRbohA* in nodule functioning. Thus, we focused our work on studying further its involvement in nodule performance.

### *MtRbohA* is linked to nodule nitrogen fixation activity

**Figure 5:**
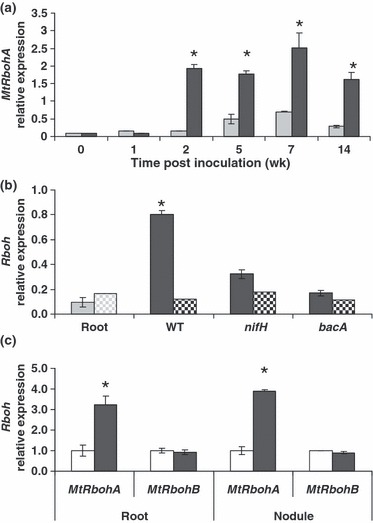
*MtRbohA* expression during nodule development. (a) Time course of *MtRbohA* expression in nodules from 1 to 14 wk post-inoculation (root, light grey bars; nodule, dark grey bars). (b) *MtRbohA* (closed bars) and *MtRbohB* (chequered bars) expression in roots (light grey bars) and nodules (dark grey bars) from 3-wk-old plants inoculated with *Sinorhizobium melilotinifH* and *bacA* mutants. (c) *MtRbohA* and *MtRbohB* relative expression levels in roots and nodules from control (open bars) or hypoxic conditions (closed bars). The value for the control condition is set to unity as reference. Values were normalized as in Fig. 2. Samples were obtained from the pooling of nodules of 20 plants and are a mean of three independent biological replicates. Asterisks (*) represent significant differences for nodules compared with roots for *P* < 0.05 (a, b) and significant differences between control and hypoxia-treated plants for *P* < 0.05 (c). Error bars, ± SE.

The expression of *MtRbohA* in nodules 7 d post-inoculation was found to be at the same level as in the roots. However, from 2 to 14 wk post-inoculation, *MtRbohA* expression was *c.* 10-fold higher in nodules compared with roots (Fig. 5a). The 7-d-old nodules are small and white, the cells are starting to be infected, but are still ineffective, unable to fix N_2_. Later, the nodules become pink, because of the presence of leghaemoglobin, an essential cytosolic oxygen transporter to the microsymbionts, and thus the nodules gain the capacity to fix N_2_ (Gage, 2004). Therefore, these results show a strong link between the nodule functionality, in terms of N_2_ fixation, and the expression of *MtRbohA*.

To further confirm this relationship, we inoculated *M. truncatula* roots with *S. meliloti* mutants unable to form functional nodules. *S. meliloti nifH* mutants are known to form *fix*^*−*^ nodules and are described as being early senescent (Hirsch *et al.*, 1983). The nodules formed by *nifH* mutants are similar in structure to the wild-type, except that *nifH* bacteroids accumulate a compact, electron-dense body (Hirsch *et al.*, 1983). In contrast with *S. meliloti nifH* mutants, *S. meliloti bacA* mutants form nodules with a disrupted structure compared with the wild-type; these nodules lack the nitrogen fixation zone, as, during the infection process, bacteria are released from the infection thread, but then undergo senescence without infecting plant cells (Glazebrook *et al.*, 1993).

The 3-wk-old nodules formed with either *S. meliloti nifH* or *bacA* mutants showed an *MtRbohA* expression level not significantly different from that of the roots, in contrast with nodules formed with *S. meliloti* wild-type bacteria which showed the already described enhanced expression (Fig. 5b). These results indicate that a nonfunctional nodule, caused either by the absence of infected cells or the inability of bacteria to fix N_2_ (as their nitrogenase complex is nonfunctional), do not show an increase in *MtRbohA* expression. By contrast, *MtRbohB* expression was not modulated in these *fix*^*−*^ phenotypes (Fig. 5b). These data further support the link between nodule functionality and *MtRbohA* expression, and point to a potential role for *MtRbohA* in nodule performance.

As the nodule fixation zone is characterized by a low oxygen tension, and as it has been proposed that NOXs may act as oxygen sensors under hypoxic conditions (Jones *et al.*, 2000; Bailey-Serres & Chang, 2005), the effect of hypoxia on *MtRbohA* expression was tested. The results (Fig. 5c) clearly show that *MtRbohA* expression is enhanced significantly under hypoxic conditions in both roots and nodules. It must be highlighted here that *MtRbohB* expression was not modulated by hypoxia, indicating that hypoxia does not result in a generalized effect on the expression of all *MtRboh* genes (Fig. 5c).

Thus, we used an RNAi approach to study the effect of a reduction in *MtRbohA* transcript levels on the ability of the nodule to fix N_2_. For this purpose, we used the *MtNCR001* promoter (Mergaert *et al.*, 2003), which expresses constitutively in the nitrogen-fixing zone, in order to drive the expression of an RNAi construct targeting the 3′-UTR of *MtRbohA*. This targeted approach to nodule functionality avoids any other collateral effect that could affect root or nodule development. An empty vector was used as a control.

**Figure 6:**
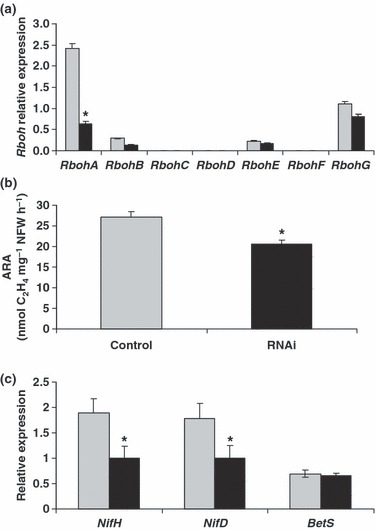
*MtRbohA* RNA interference (RNAi) phenotype. Relative *MtRboh* gene expression (a), nitrogen fixation activity (b) and relative *Sinorhizobium meliloti* nitrogenase gene expression (c) were obtained from the pooling of controls (empty vector) or *MtRbohA* RNAi composite plants (*n* > 40). Control, light grey bars; RNAi, black bars. Values are representative of three independent biological replicates. Gene expression values were normalized against *40S Ribosomal Protein S8, Mtc27* (*Medicago truncatula*) and *smc00324* (*S. meliloti*) genes. ARA, acetylene reduction assay; NFW, nodule fresh weight. Asterisk (*) represents significant differences compared with control plants for *P* < 0.05. Error bars, ± SE.

The RNAi construct led to a reduction of > 60% in the *MtRbohA* mRNA level in nodules (compared with control transgenic nodules), whereas no effect was detected on other *MtRboh* gene expression (Fig. 6a). The decrease in mRNA level provoked a 25% reduction in nodule nitrogen fixation activity (Fig. 6b), which was not related to nodule fresh weight as that did not vary (12.3 ± 1.2 and 12.2 ± 0.9 mg of nodule fresh weight per plant for controls and RNAi lines, respectively). In addition, no difference was observed either macroscopically or by light microscopy in nodule structure that could explain the depletion of nitrogen fixation in RNAi lines (Fig. S4). Thus, this phenotype may be caused by an effect on nodule metabolism rather than to a disruption of nodule structure. It should be noted that similar results were obtained with the constitutive 35S promoter (Fig. S5); no other phenotype was observed in these plants.

The expression levels of plant and bacterial genes known to be involved in nodule functioning were tested in nodules formed on roots transformed with the *MtRbohA* RNAi construct or with the control vector. Plant genes encoding sucrose synthase (*MtSucS1*; *Medtr8g133160.1*), glutamine synthase 1a (*Medtr6g080780.1*), phosphoenol pyruvate carboxylase (*Medtr2g092340.1*) and phosphoenol pyruvate carboxylase kinase (*Medtr1g093530.1*), which play major roles in nodule functioning (Carvalho *et al.*, 2003; Nomura *et al.*, 2006; Baier *et al.*, 2007; Xu *et al.*, 2007), did not appear to show modulated expression (data not shown). By contrast, the expression of the microsymbiont *NifD* and *NifH* genes was decreased significantly in nodules formed on *MtRbohA* transgenic roots (Fig. 6c), whereas the expression of other genes – for example *BetS*, which is involved in osmotic protection (Boscari *et al.*, 2006) – was not affected (Fig. 6c).

## Discussion

### *Rboh* genes in the *M. truncatula* genome

The aim of our work was to evaluate the involvement of *M. truncatula* RBOH proteins during its symbiotic interaction with *S. meliloti*. Using a sequence similarity search, we were able to identify seven genes encoding MtRBOH proteins in the incomplete *M. truncatula* genome with identity levels ranging from 47% to 69%. Based on data from already sequenced higher plant genomes and ESTs, other *Rboh* genes should be present in the *M. truncatula* genome. Indeed, the presence of four ESTs (EST642356, EST396294, EST317358, TC116307) matching *Rboh* sequences distinct from *MtRbohA–G* clearly suggests a larger number of *Rboh* genes in the *M. truncatula* genome. Interestingly, legume-specific duplications of *Rboh* genes, encompassing those of *M. truncatula*, can be deduced from the phylogeny in groups 1 and 2. In group 2, the duplications preceded the separation of the different legume species and continued independently in *M. truncatula* and *G. max*, but apparently not in *L. japonicus*. This tendency is particularly exemplified by the *MtRbohB–D* genes, which are all co-orthologous to *Glyma04g38040* and *Glyma06g17030* genes. These three *M. truncatula* genes are localized in chromosome 3 within 40 kbp, suggesting tandem duplications. In group 1, duplications also appear to have preceded the speciation of the different legume species, but apparently pursued only in *L. japonicus* and *G. max* (Schmutz *et al.*, 2010). Overall, these legume-specific duplications may have allowed functional divergence or the emergence of new function. Interestingly, all Arabidopsis genes have orthologues in legumes, except *AtRbohD*, which was either lost in legumes or has not yet been identified in these plants.

### *MtRboh* genes during root and nodule development

The connection between ROS formation, root development and physiological processes has already been highlighted (Joo *et al.*, 2001; Liszkay *et al.*, 2004; Su *et al.*, 2006; Li *et al.*, 2009). Nevertheless, little information is available on ROS generation related to root growth. The very high *MtRboh* expression level in the meristematic and elongation zones of the root, shown in Fig. 3, suggests the involvement of NOXs during root growth, where they may also play a role in cell wall expansion (Monshausen *et al.*, 2007; Macpherson *et al.*, 2008). Furthermore, the high *MtRbohF* expression in developing root hairs is in line with previous results showing that ROS accumulate in growing root hairs (Foreman *et al.*, 2003), and that blocking of the activity of NOXs with DPI inhibits ROS formation and affects root hair growth (Foreman *et al.*, 2003; Cardenas *et al.*, 2008). Thus, one can suggest that *MtRbohF* could play a role in *M. truncatula* root hair development. This would be in agreement with the concept of ROS production by plasma membrane RBOHs being a general mechanism in the control of the polarized growth of plant cells (Liu *et al.*, 2009).

The expression of *MtRboh* genes in the meristematic zone and vascular tissues of the root nodule, on symbiotic interaction with *S. meliloti* (Fig. 4), is in agreement with the detection of O_2_^−^ and H_2_O_2_ in the nodule cortex and meristematic cells (Groten *et al.*, 2005; Rubio *et al.*, 2009). This points to a role for NOXs in nodule development. It must be underlined here that ROS produced by a fungal NOX (*NoxA*) regulate hyphal growth in the mutualistic interaction between a fungal endophyte and its grass host (Tanaka *et al.*, 2006). Moreover, a regulator of *NoxA* is essential *in planta* for the symbiotic interaction (Takemoto *et al.*, 2006). Taken together, these data suggest that RBOHs are required for the optimal establishment of fungal (Scott & Eaton, 2008) and rhizobial symbioses.

Interestingly, none of the analysed *MtRboh* promoters yielded GUS staining in the senescence zone (Fig. 4). Nodule senescence is an active process programmed in development, in which ROS, antioxidants, hormones and proteinases have a key role (Puppo *et al.*, 2005). On the other hand, several reports have described ROS and RBOH involvement during programmed cell death during plant–pathogen interactions (Torres *et al.*, 2002; Torres & Dangl, 2005). Our results suggest that these *MtRboh* genes do not appear to be involved in nodule senescence. Therefore, these results are in agreement with the hypothesis proposed by Puppo *et al.* (2005) involving ROS in nodule senescence related to a progressive decline in antioxidant content (ascorbate and glutathione), rather than to an increase in ROS production itself.

### *MtRbohA* and nodule functioning

Our results indicate that *MtRbohA* is involved in nodule functioning. Indeed, the *MtRbohA* expression level appeared to be concomitant with the establishment of a functioning nodule, as from 2 wk post-inoculation with *S. meliloti*, *MtRbohA* expression was *c.* 10-fold higher in nodules than in roots, whereas, in very young nodules, its expression was at the same level as in the roots (Fig. 5a). Moreover, this was not observed when the inoculation was performed with rhizobial mutants unable to form functional nodules (Fig. 5b). In the same way, the decrease in *MtRbohA* expression *via* the RNAi approach led to a reduction in the nitrogen fixation capacity (Fig. 6b), again showing the link between MtRBOHA and nodule functionality. The down-regulation of the microsymbiont *nifD* and *nifH* genes may contribute to an explanation of the decrease in nitrogen fixation activity. Indeed, these genes encode the Mo–Fe and Fe proteins of the nitrogenase complex, respectively, which is responsible for dinitrogen reduction into ammonia. This may indicate that MtRBOHA activity contributes to the communication between the plant and the endosymbiont. Again, this is reminiscent of the important role played by NOXs in the establishment of some beneficial plant–microbe interactions (Takemoto *et al.*, 2006; Tanaka *et al.*, 2006).

However, the absence of any effect of the 35S construct on either the kinetics or intensity of the nodulation process indicates that *MtRbohA* does not play a role in the early steps of the symbiotic interaction, thus excluding this isoform as a candidate for ROS production at this stage (Santos *et al.*, 2001; Ramu *et al.*, 2002).

Moreover, *MtRbohA* expression appears to be largely increased under hypoxic conditions (Fig. 5c). Similarly, the nitrogen-fixing zone has a microaerobic environment, allowing the functioning of the microsymbiont nitrogenase. Although the possible role of NOXs in the oxygen-sensing processes has been suggested (Jones *et al.*, 2000; Bailey-Serres & Chang, 2005), they do not appear to have redox centres that are oxidized/reduced in response to oxygen. Thus, the cascade of events could be as follows: the hypoxia-driven stimulation of *MtRbohA* expression would, in turn, lead to the regulation of the expression of genes and/or to post-translational modifications involved in nodule functioning.

In conclusion, the results presented in this report shed new light on the role(s) of RBOHs in plant–microbe interactions. Until now, their roles have been essentially, if not exclusively, linked to plant defence reactions against invading microbes in incompatible reactions. We have shown, in particular, that at least one RBOH may be necessary for optimal functioning of the *M. truncatula–S. meliloti* nodule. Future work will aim at studying the involvement of other(s) *MtRboh* gene(s) in the symbiotic process. Moreover, the identification of *MtRbohA* molecular targets in both partners will help to elucidate its role in plant–microsymbiont communication.

## Supporting Information

Fig. S1 Bayesian phylogenetic tree of respiratory burst oxidase homologue (RBOH) amino acid sequences in Viridiplantae. Fig. S2 *MtRboh* gene expression analysis in different plant tissues. Fig. S3 Simultaneous *MtRbohA*expression and *Sinorhizobium meliloti* localization in nodules. Fig. S4 Morphological analysis of controland *MtRbohA* RNAi nodules. Fig. S5 Nitrogen fixation activity in3SS::*MtRbohA* RNAi nodules.

Table S1 Respiratory burst oxidase homologue (RBOH) protein sequences used for the phylogenetic analysis Table S2 Primers used for quantitativereal-time PCR analysis and for *MtRboh* promoter cloning Table S3 MtRBOH protein sequence similarities
