# Supplementary material for: A Medicago truncatula NADPH oxidase is involved in symbiotic nodule functioning
Source: New Phytol. 2011 Jan;189(2):580–92. doi: 10.1111/j.1469-8137.2010.03509.x (PMC3491693; doi:10.1111/j.1469-8137.2010.03509.x)
Supplement: Supplementary file 1 — Fig. S1 Bayesian phylogenetic tree of respiratory burst oxidase homologue (RBOH) amino acid sequences in Viridiplantae. Fig. S2 MtRboh gene expression analysis in different plant tissues. Fig. S3 Simultaneous MtRbohAexpression and Sinorhizobium meliloti localization in nodules. Fig. S4 Morphological analysis of controland MtRbohA RNAi nodules. Fig. S5 Nitrogen fixation activity in3SS::MtRbohA RNAi nodules. [file nph0189-0580-SD1.ppt]

## Slide 1
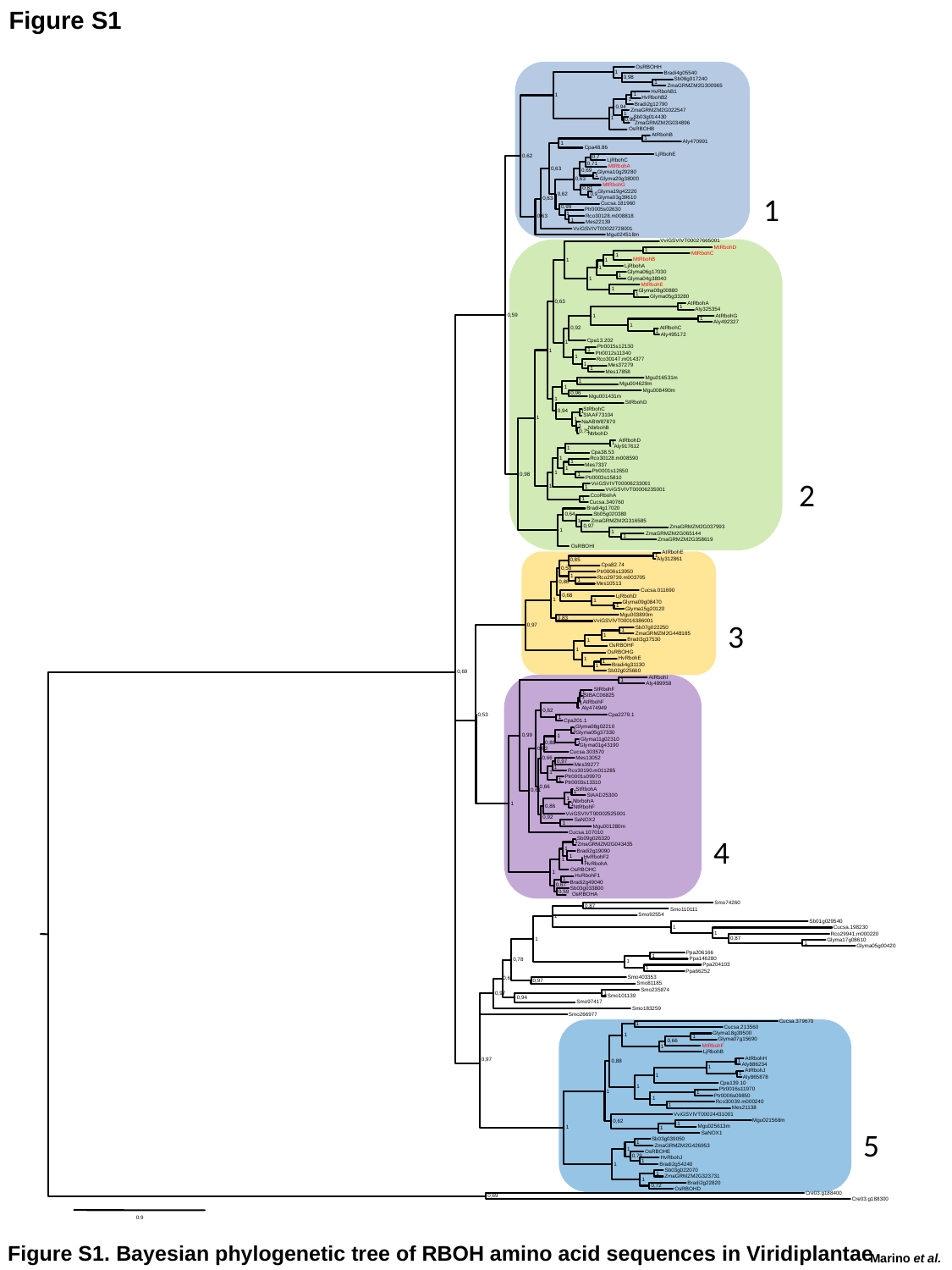

Figure S1
OsRBOHH
1
Bradi4g05540
0,98
Sb08g017240
1
ZmaGRMZM2G300965
HvRbohB1
1
1
HvRbohB2
1
Bradi2g12790
0,94
ZmaGRMZM2G022547
1
Sb03g014430
1
0,99
ZmaGRMZM2G034896
OsRBOHB
AtRbohB
1
Aly470991
1
Cpa48.86
LjRbohE
0,62
0,7
LjRbohC
0,71
MtRbohA
0,63
0,69
Glyma10g29280
1
Glyma20g38000
0,63
MtRbohG
0,81
1
2
3
4
5
Glyma19g42220
0,62
0,9
Glyma03g39610
0,63
Cucsa.181960
0,99
Ptr0005s02630
1
Rco30128.m008818
0,63
1
Mes22139
VviGSVIVT00022728001
Mgu024518m
VviGSVIVT00027665001
MtRbohD
1
MtRbohC
1
MtRbohB
1
1
LjRbohA
1
Glyma06g17030
1
Glyma04g38040
1
MtRbohE
1
Glyma08g00880
1
Glyma05g33280
0,63
AtRbohA
1
Aly325354
0,59
1
AtRbohG
1
Aly492327
1
0,92
AtRbohC
1
Aly495172
Cpa13.202
1
Ptr0015s12130
1
1
Ptr0012s11340
1
Rco30147.m014377
1
Mes37279
1
Mes17858
Mgu016531m
1
Mgu004629m
1
Mgu008490m
0,96
Mgu001431m
1
StRbohD
StRbohC
0,94
1
SlAAF73104
1
1
NaABW87870
1
NbrbohB
0,75
NtrbohD
AtRbohD
1
Aly917612
1
Cpa38.53
1
Rco30128.m008590
1
Mes7337
1
Ptr0001s12650
1
0,98
1
Ptr0003s15810
VviGSVIVT00006233001
1
1
VviGSVIVT00006235001
CcoRbohA
1
Cucsa.340760
Bradi4g17020
0,64
Sb05g020380
1
ZmaGRMZM2G316585
0,97
ZmaGRMZM2G037993
1
1
ZmaGRMZM2G065144
1
ZmaGRMZM2G358619
OsRBOHI
AtRbohE
1
Aly312861
0,85
Cpa82.74
0,58
Ptr0006s13950
1
Rco29739.m003705
1
0,86
Mes10513
Cucsa.011690
0,68
LjRbohD
1
1
Glyma09g08470
1
Glyma15g20120
Mgu003890m
0,83
VviGSVIVT00016386001
0,97
Sb07g022250
1
ZmaGRMZM2G448185
1
Bradi3g37530
1
OsRBOHF
1
OsRBOHG
HvRbohE
1
1
Bradi4g31130
1
Sb02g025660
0,69
AtRbohI
1
Aly489958
StRbohF
1
StBAC06825
1
AtRbohF
1
Aly474949
0,62
0,53
Cpa2279.1
1
Cpa201.1
Glyma08g02210
1
Glyma05g37330
0,99
1
Glyma11g02310
1
0,88
Glyma01g43190
0,62
Cucsa.303570
Mes13052
0,66
0,97
Mes39277
1
Rco30190.m011285
1
Ptr0001s09970
1
Ptr0003s13310
0,66
StRbohA
0,61
1
SlAAD25300
1
NbrbohA
1
1
0,86
NtRbohF
VviGSVIVT00002525001
0,92
SaNOX2
1
Mgu001280m
Cucsa.107010
Sb09g026320
1
ZmaGRMZM2G043435
1
Bradi2g19090
1
HvRbohF2
1
1
HvRbohA
OsRBOHC
1
HvRbohF1
1
Bradi2g49040
0,67
Sb03g033800
0,59
OsRBOHA
Smo74260
0,87
Smo110111
Smo92554
1
Sb01g029540
1
Cucsa.198230
1
Rco29941.m000220
0,87
1
Glyma17g08610
1
Glyma05g00420
Ppa206166
1
Ppa146280
0,78
1
Ppa204103
1
Ppa66252
Smo403353
0,6
0,97
Smo81185
Smo235874
1
0,97
Smo101139
0,94
Smo97417
Smo183259
Smo266977
Cucsa.379670
1
Cucsa.213560
Glyma18g39500
1
1
Glyma07g15690
0,66
MtRbohF
1
LjRbohB
AtRbohH
0,97
0,88
1
Aly886234
1
AtRbohJ
1
1
Aly865878
Cpa139.10
1
Ptr0016s11970
1
1
Ptr0006s09850
1
Rco30039.m000240
1
Mes21138
VviGSVIVT00024431001
Mgu021568m
0,62
1
Mgu025613m
1
1
SaNOX1
Sb03g039050
1
ZmaGRMZM2G426953
1
OsRBOHE
0,79
HvRbohJ
1
Bradi2g54240
1
Sb03g022070
1
ZmaGRMZM2G323731
1
Bradi2g22820
0,72
OsRBOHD
Cre03.g188400
0,69
Cre03.g188300
0.9
Figure S1. Bayesian phylogenetic tree of RBOH amino acid sequences in Viridiplantae
Marino et al.

## Slide 2
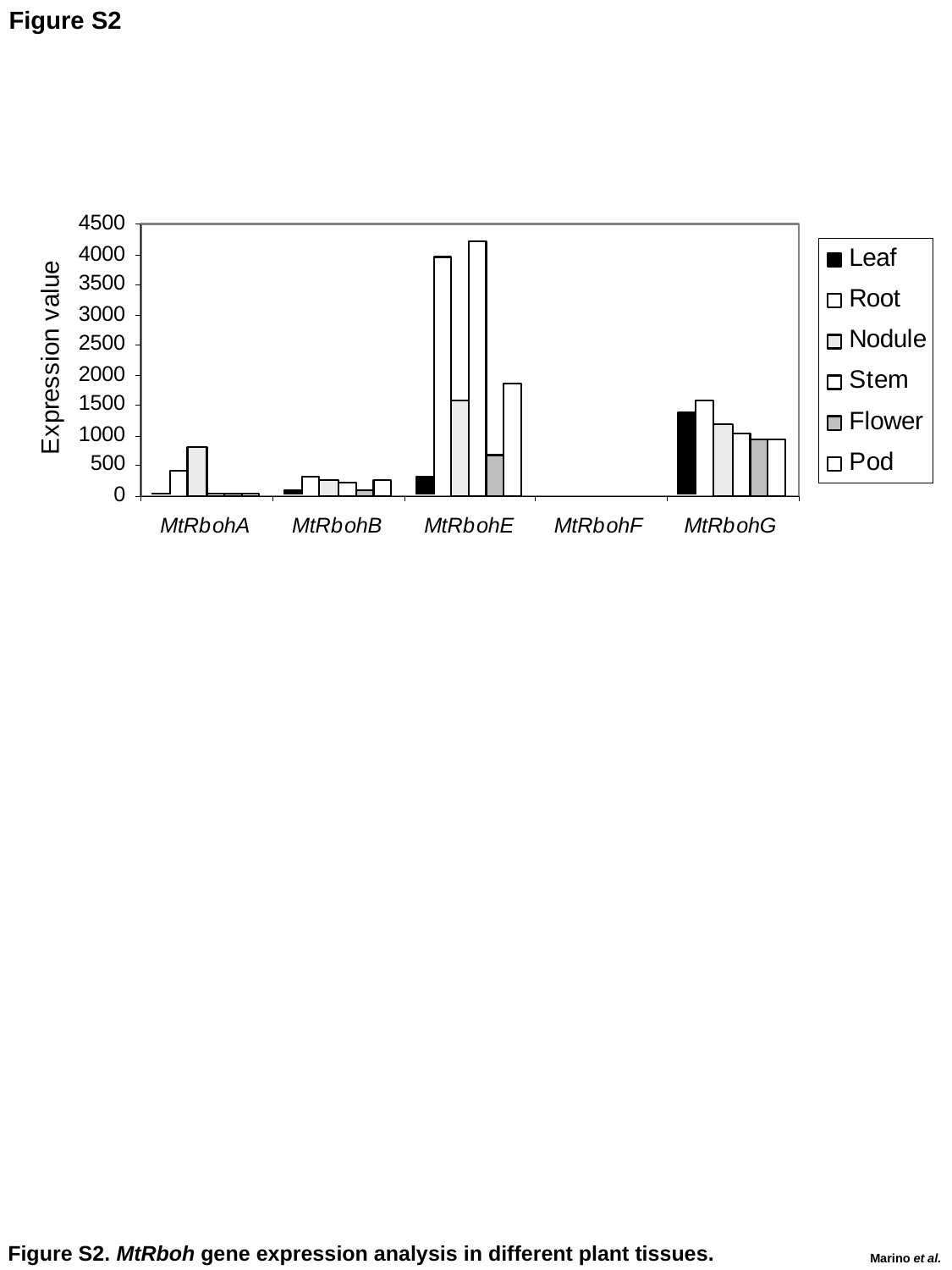

Figure S2
Figure S2. MtRboh gene expression analysis in different plant tissues.
Marino et al.

## Slide 3
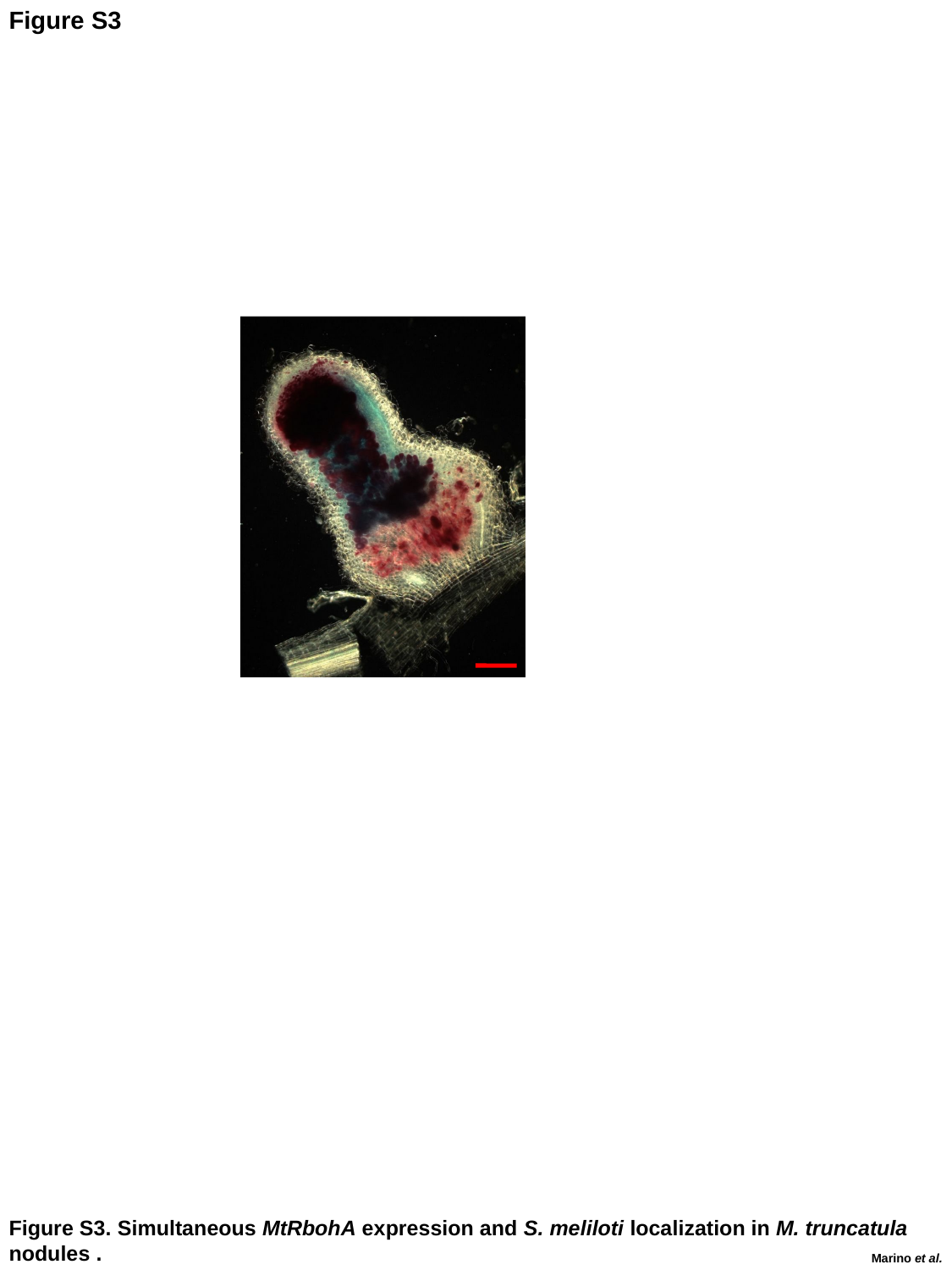

Figure S3
Figure S3. Simultaneous MtRbohA expression and S. meliloti localization in M. truncatula nodules .
Marino et al.

## Slide 4
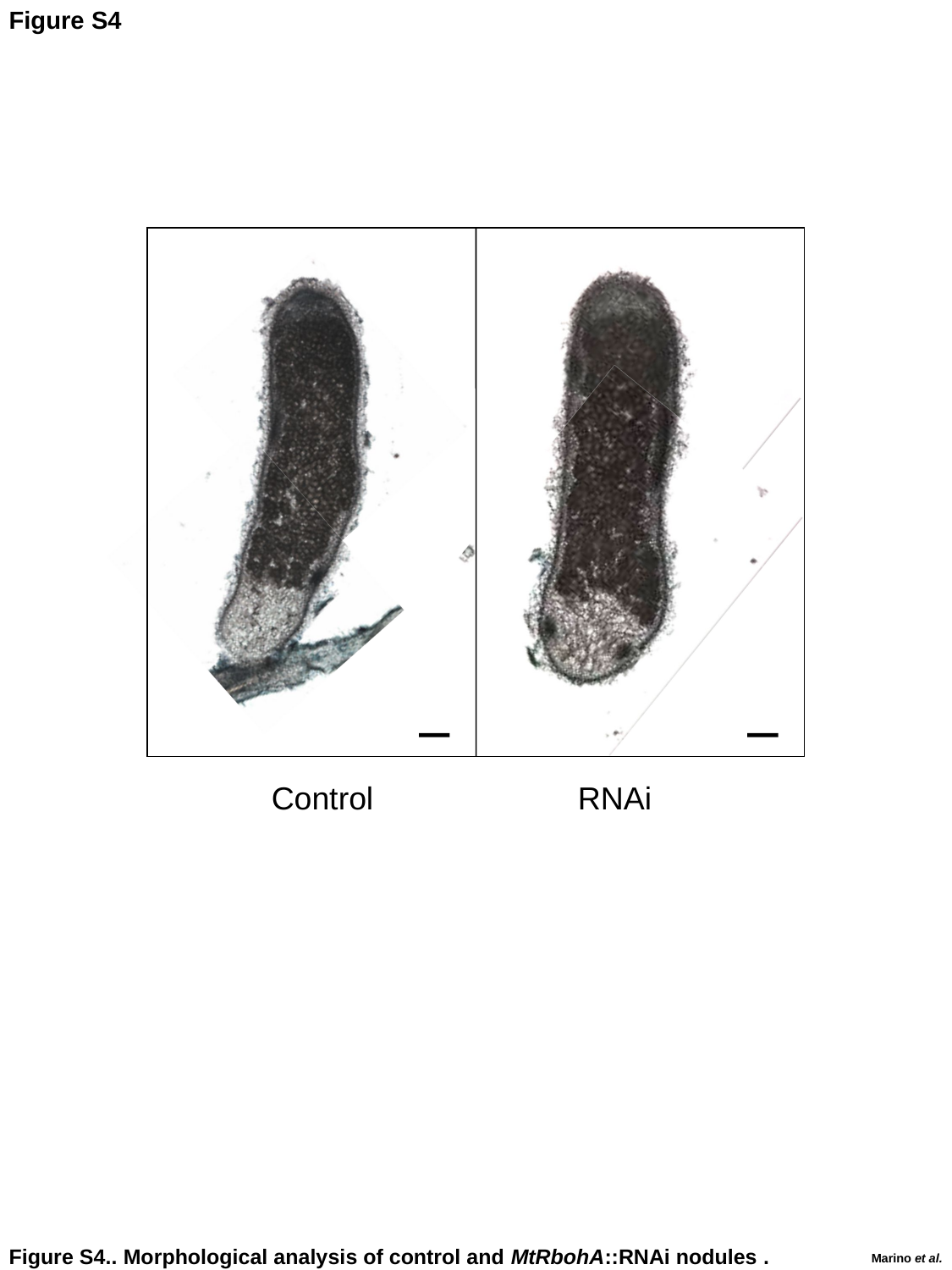

Figure S4
Control
RNAi
Figure S4.. Morphological analysis of control and MtRbohA::RNAi nodules .
Marino et al.

## Slide 5
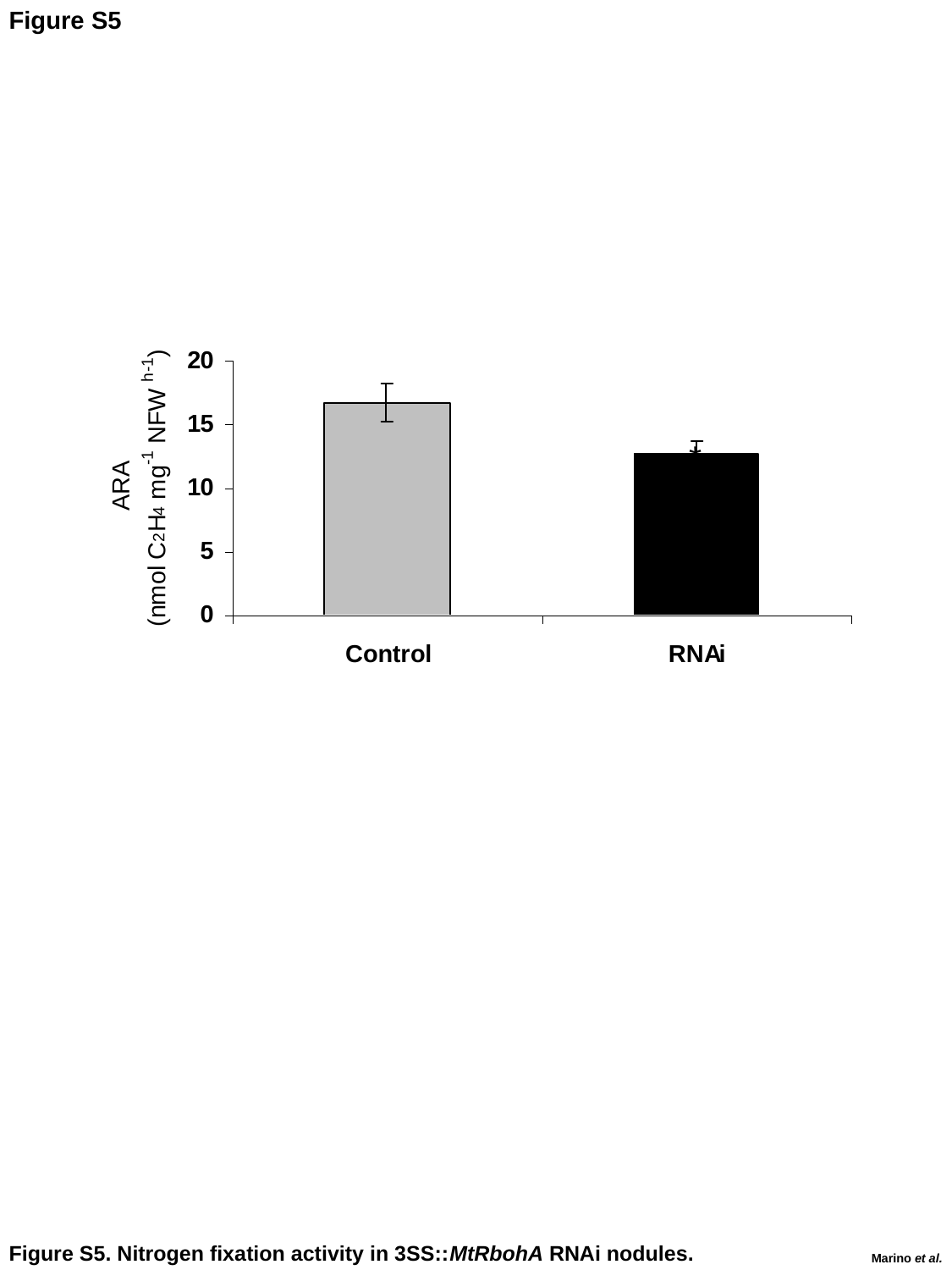

Figure S5
*
Figure S5. Nitrogen fixation activity in 3SS::MtRbohA RNAi nodules.
Marino et al.
