# Supplementary material for: A Medicago truncatula NADPH oxidase is involved in symbiotic nodule functioning
Source: New Phytol. 2011 Jan;189(2):580–92. doi: 10.1111/j.1469-8137.2010.03509.x (PMC3491693; doi:10.1111/j.1469-8137.2010.03509.x)
Supplement: Supplementary file 2 — Fig. S1 Bayesian phylogenetic tree of respiratory burst oxidase homologue (RBOH) amino acid sequences in Viridiplantae. Fig. S2 MtRboh gene expression analysis in different plant tissues. Fig. S3 Simultaneous MtRbohAexpression and Sinorhizobium meliloti localization in nodules. Fig. S4 Morphological analysis of controland MtRbohA RNAi nodules. Fig. S5 Nitrogen fixation activity in3SS::MtRbohA RNAi nodules. [file nph0189-0580-SD2.doc]

**Supporting Information Legends (Marino *et al*.): Figs S1–S5**

**Figure S1. Bayesian phylogenetic tree of RBOH amino acid sequences in Viridiplantae.** Twenty seven plant species with a whole or partial genome publicly available were used for the analysis. Species name abbreviations are used as prefix as follows; Al stands for *Arabidopsis lyrata*, At for *Arabidopsis thaliana*, Bradi for *Brachypodium distachyon*, Cco for *Citrullus colocynthis*, Cpa for *Carica papaya*, Cre for *Chlamydomonas reinhardtii*, Cusca for *Cucumis sativus*, Glyma stands for *Glycine max*, Hv for *Hordeum vulgare*, Lj for *Lotus japonicas*, Mes for *Manihot esculenta*, Mgu for *Mimulus guttatus*, Mt for *Medicago truncatula*, Na for *Nicotiana attenuata*, Nb for *Nicotiana benthamiana*, Nt for *Nicotiana tabacum*, Os for *Oryza sativa*, Ppa for *Physcomitrella patens*, Ptr for *Populus trichocarpa*, Rco for *Ricinus communis*, Sa for *Striga asiatica*, Sb for Sorghum bicolor, Sl for *Solanum lycopersicum*, Smo for *Selaginella moellendorffii*, St for *Solanum tuberosum*, Vvi for *Vitis vinifera* and Zma for *Zea mays*. This Bayesian tree topology was obtained after 800,000 generations (necessary to reach congruence) with MrBayes using a mixture of models. The model with the best posterior probability (PP) was the Jones model (PP=1.0), the estimated proportion of invariable site was 0.019 and the alpha parameter of the shape of the gamma distribution was 0.823. The phylogenetic tree was represented as a phylogram and midpoint-rooted. Posterior probability support values are indicated at each node. *M. truncatula* *Rboh* genes are highlighted in red. Phylogenetic groups 1 to 5 are represented by colored rounded-rectangles.

**Figure S2. *MtRboh* gene expression analysis in different plant tissues.** Data were imported from the *Medicago truncatula* Gene Expression Atlas using the Affymetrix probeset listed in table S2.

**Figure S3. Simultaneous *MtRbohA* expression and *S. meliloti* localization in *M. truncatula* nodules.** Four week-old nodules from composite plants expressing p*RbohA*::GUS transcriptional fusion inoculated with a *S. meliloti* *hemA:lacZ* strain were stained for GUS and LacZ activity, respectively. To differentiate the lacZ staining from the GUS one, Salmon-gal (Fluka, [www.sigmaaldrich.com](http://www.sigmaaldrich.com/)) was used as a substrate to give a red staining to *S. meliloti* cells. Scale bars represent 200 µm. n = 10.

**Figure S4. Morphological analysis of control and *MtRbohA* RNAi nodules.** Longitudinal sections (100 µm) from control and *MtRbohA* RNAi four week-old nodules were visualized with a microscope. Scale bars represent 200 µm. n = 10.

**Figure S5. Nitrogen fixation activity in 3SS::*MtRbohA* RNAi nodules.** Nitrogen fixation activity were obtained from pooling controls (empty vector) or 35S::*MtRbohA* RNAi composite plants (n>30). ARA: Acetylene reduction assay. Asterisk (*) represents significant differences compared to control plants for p<0.05. Error bars represent standard errors.
