# Supplementary material for: A Medicago truncatula NADPH oxidase is involved in symbiotic nodule functioning
Source: New Phytol. 2011 Jan;189(2):580–92. doi: 10.1111/j.1469-8137.2010.03509.x (PMC3491693; doi:10.1111/j.1469-8137.2010.03509.x)
Supplement: Supplementary file 3 [file nph0189-0580-SD3.doc]

**Table S1: RBOH protein sequences used for** the phylogenetic analysis.

| **Species** | **Classification ^a^** | **Accession number/gene ID** |
| --- | --- | --- |
| *Arabidopsis thaliana* | Eudicots/eurosids II | AtRBOHA-J^b^ |
| *Arabidopsis lyrata* | Eudicots/eurosids II | Aly495172, Aly865878, Aly474949, Aly489958, Aly325354, Aly470991, Aly312861, Aly886234, Aly492327, Aly917612^c^ |
| *Brachypodium distachyon* | Monocots | Bradi2g12790, Bradi2g19090, Bradi2g22820, Bradi2g49040, Bradi2g54240, Bradi3g37530, Bradi4g05540, Bradi4g17020, Bradi4g31130^c^ |
| *Carica papaya* |  | Cpa13.202, Cpa139.10, Cpa201.1, Cpa2279.1, Cpa38.53, Cpa48.86, Cpa82.74^c^ |
| *Chlamydomonas reinhardtii* | Chlorophyta | Cre03.g188400, Cre03.g188300^c^ |
| *Citrullus colocynthis* | Eudicots/eurosids I | ACF05504.2/CcoRbohA^b^ |
| *Cucumis sativus* | Eudicots/eurosids I | Cucsa.011690, Cucsa.107010, Cucsa.181960, Cucsa.198230, Cucsa.213560, Cucsa.303570, Cucsa.340760, Cucsa.379670^c^ |
| *Glycine max* | Eudicots/eurosids I | Glyma08g00880, Glyma19g42220, Glyma06g17030, Glyma04g38040, Glyma11g02310, Glyma10g29280, Glyma03g39610, Glyma20g38000, Glyma18g39500, Glyma01g43190, Glyma07g15690, Glyma08g02210, Glyma09g08470, Glyma15g20120, Glyma17g08610, Glyma05g37330, Glyma05g33280, Glyma05g00420^c^ |
| *Hordeum vulgare* | Monocots | HvRBOHA, B1, B2, E, F1, F2, J^b^ |
| *Lotus japonicus* | Eudicots/eurosids I | chr5.CM0299.380.nc (LjRBOHC); chr5.CM0299.370.nc (LjRBOHE); chr1.CM0094.200.nc (LjRBOHA), chr6.CM0013.340.nc (LjRBOHD); chr1.CM0147.340.nc (LjRBOHB)^d^ |
| *Manihot esculenta* | Eudicots/eurosids I | Mes13052, Mes39277, Mes37279, Mes21138, Mes10513, Mes17858, Mes7337, Mes22139^c^ |
| *Medicago truncatula* | Eudicots/eurosids I | see table x |
| *Mimulus guttatus* | Euasterid I | Mgu016531m, Mgu001431m, Mgu001280m, Mgu004629m, Mgu025613m, Mgu008490m, Mgu021568m, Mgu024518m, Mgu003890m^c^ |
| *Nicotiana attenuata* | Euasterid I | ABW87870.1^b^ |
| *Nicotiana benthamiana* | Euasterid I | BAC56864.1 (NbRBOHA), BAC56865.1 (NbRBOHB)^b^ |
| *Nicotiana tabacum* | Euasterid I | CAC84140.1 (NtRBOHD), ABS85195.1 (NtRBOHF)^b^ |
| *Oryza sativa* | Monocots | Os01g25820, Os01g61880, Os05g38980, Os05g45210, Os08g35210, Os09g26660, Os12g35610, Os11g33120, Os01g53294^c^ |
| *Physcomitrella patens* | Bryophyta | Ppa204103, Ppa66252, Ppa206166, Ppa146280^c^ |
| *Populus trichocarpa* | Eudicots/eurosids I | Ptr0005s02630, Ptr0006s13950, Ptr0015s12130, Ptr0012s11340, Ptr0001s12650, Ptr0003s15810, Ptr0016s11970, Ptr0001s09970, Ptr0003s13310, Ptr0006s09850^c^ |
| *Ricinus communis* | Eudicots/eurosids I | Rco29739.m003705, Rco29941.m000220, Rco30039.m000240, Rco30128.m008590, Rco30128.m008818, Rco30147.m014377, Rco30190.m011285^c^ |
| *Selaginella moellendorffii* | Lycophyta | Smo110111, Smo81185, Smo403353, Smo74260, Smo235874, Smo97417, Smo266977, Smo92554, Smo183259, Smo101139^c^ |
| *Solanum lycopersicum* | Euasterid I | AAF73104, AAD25300^b^ |
| *Solanum tuberosum* | Euasterid I | Q948U0.1 (StRBOHA), Q2HXL0.2 (StRBOHC), Q2HXK9.2 (StRBOHD), BAB84124.1 (StRBOHF), BAC06825 (StBAC06825)^b^ |
| *Sorghum bicolor* | Monocots | Sb01g029540, Sb02g025660, Sb03g014430, Sb03g022070, Sb03g033800, Sb05g020380, Sb07g022250, Sb08g017240, Sb09g026320, Sb03g039050^c^ |
| *Striga asiatica* | Euasterid I | ABG35770.1 (SaNOX1), ABG35769.1 (SaNOX2)^b^ |
| *Vitis vinifera* | Eudicots/rosids | VviGSVIVT00002525001, VviGSVIVT00006233001, VviGSVIVT00006235001, VviGSVIVT00016386001, VviGSVIVT00022728001, VviGSVIVT00024431001, VviGSVIVT00027665001^c^ |
| *Zea mays* | Monocots | ZmaGRMZM2G022547, ZmaGRMZM2G034896, ZmaGRMZM2G037993, ZmaGRMZM2G043435, ZmaGRMZM2G065144, ZmaGRMZM2G300965, ZmaGRMZM2G316585, ZmaGRMZM2G323731, ZmaGRMZM2G358619, ZmaGRMZM2G426953, ZmaGRMZM2G448185^c^ |

^a^Plant classification refers to APGII: [www.mobot.org/MOBOT/Research/APweb/welcome.html](http://www.mobot.org/MOBOT/Research/APweb/welcome.html)

^b^Gene ID obtained from <http://www.ncbi.nlm.nih.gov/genbank>

^c^Gene ID obtained from [http://www.phytozome.net](http://www.phytozome.net/)

^d^Gene ID obtained from [www.kazusa.or.jp/lotus](http://www.kazusa.or.jp/lotus)

**Table S2. Primers used for qRT-PCR analysis and for *MtRboh* promoters cloning.** For *MtRboh* promoter primers an *AttB* recombination sequence adaptor was added for cloning *via* Gateway technology (Invitrogen, [www.invitrogen.com](http://www.invitrogen.com/)).

|  | ***Target*** | ***Forward (5’ → 3’)*** | ***Reverse (5’ → 3’)*** | ***Amplicon (bp)*** |
| --- | --- | --- | --- | --- |
|  | *MtRbohA* | GCGCAACTCCTTTGATTAGC | GAAATAGGCTCGCTTGGTTG | 143 |
|  | *MtRbohB* | GCTCGCTCTGCTCTTATTGC | TGCGCTTGTAGACACTACGC | 127 |
|  | *MtRbohC* | GGGAGACCTGATGCTATTCAAG | TGTCTTCAACAATAATGTCCATCTG | 122 |
|  | *MtRbohD* | ACATGGCTCAGGAGCAAGAC | TGAAGAAGGCGTGGAAAGTC | 149 |
| **qRT-PCR** | *MtRbohE* | CCAAATTCTCACAGGCTTGC | CTCCATACGGACCATCAATC | 126 |
|  | *MtRbohF* | GAAGAGCCTTTGTCAAGAGTTTAG | GGATTGATGCTTTAGCTGTGTG | 108 |
|  | *MtRbohG* | GTGATGGATGAAATCGCAGA | TGGCATGATGAAGTGATTGA | 130 |
|  | *SmbetS* | AAATCCACCATGCCCAGTAG | CTGCCAGAACATCGGTCTCT | 119 |
|  | *MtPdc1* | CGGAGGAAGATTTGATAGAAGC | TGCTGGTGTCATCCTTGTGT | 100 |
|  | *MtSucS1* | ATTCAACTTGTAGGTCTCGAT | GTTGAGAATGAAGAGCACATA | 246 |
|  | *PEPC1* | ACTTGAAGCAAAGACTCAGACTC | CGCACCTCCACCTTGTAGTT | 109 |
|  | *PEPCK* | GTTGAGGAAGATGATGTGTAGAG | CAGATTCATTGCTTCACCTCC | 100 |
|  | *40S* | AAGAAGGGAAAGAGTGCTGCT | GGTGTCCAATTAACACTCAACC | 131 |
|  | *Mtc27* | TGAGGGAGCAACCAAATACC | GCGAAAACCAAGCTACCATC | 93 |
|  | *MtRbohA* | CAGTTTTCGGAGGTTACATCA | AGGTGATTTTTATATAATTAAGAG | 1987 |
|  | *MtRbohB* | GGTCAGCAAAGTGCTCAAC | GTTCCATGATAACTTTGGGAC | 1765 |
| **Promoter** | *MtRbohE* | GGTGGCTTTGGAGATAAAG | ACCGGAAGAATGAATGCATC | 1802 |
| **cloning** | *MtRbohF* | AAGCCGGAAAGACTTTGCTT | CAAAGGGTATTAGAGGGAAAGATG | 1722 |
|  | *MtRbohG* | ATGTCAAACCGTCGAGAAAG | GTCAAAGATGGAGGCATGAG | 1754 |

**Table S3. MtRBOH protein sequence similarities.** Values (corresponding to the percentage of similarity) were obtained by pairwise alignment using BLAST bl2seq ([www.ncbi.com](http://www.ncbi.com/)).

|  | **MtRBOHB** | **MtRBOHC** | **MtRBOHD** | **MtRBOHE** | **MtRBOHF** | **MtRBOHG** |
| --- | --- | --- | --- | --- | --- | --- |
| **MtRBOHA** | 59 | 54 | 53 | 58 | 54 | 83 |
| **MtRBOHB** | x | 69 | 65 | 69 | 51 | 61 |
| **MtRBOHC** |  | x | 69 | 58 | 46 | 55 |
| **MtRBOHD** |  |  | x | 55 | 44 | 54 |
| **MtRBOHE** |  |  |  | x | 47 | 59 |
| **MtRBOHF** |  |  |  |  | x | 54 |
| **MtRBOHG** |  |  |  |  |  | x |
